# Epididymal Metastasis from Carcinoma of the Prostate

**DOI:** 10.1100/tsw.2004.61

**Published:** 2004-06-07

**Authors:** C. Heman-Ackah, Dip Urol, A. T. Fox, J. El-Jabbour

**Affiliations:** Barnet General Hospital, Wellhouse Lane, Barnet, Herts EN5 3DJ, UK

## Abstract

Epididymal Metastasis from a primary carcinoma of the prostate gland is a rare but recognised phenomena. We describe a case of such metastasis which, unlike previous reports, presents as a painful epididymal mass. Therefore it is important for urologists to consider epididymal metastasis as part of the differential diagnosis in a patient with known carcinoma of the prostate and a tender epididymal mass.

## CASE REPORT

A 77-year-old man was initially diagnosed as having a localised carcinoma of the prostate and treated with radiotherapy. Three years later he was commenced on Cyproterone Acetate on account of a rising PSA and increasing bladder outflow obstruction. He had a good symptomatic response and his PSA fell from 86ng/ml to 8.7ng/ml within 4 months.

He remained well for fourteen months when he developed a tender lump in his left epididymis. An ultrasound scan reported normal testis with small hydroceles bilaterally and a 9mm left epididymal cyst. On examination he had a hard, tender, craggy mass in the head of his left epididymis, inconsistent with the ultrasonographic findings. Rectal examination revealed a flat, firm prostate. His PSA was 23 ng/ml. A differential diagnosis of an inflammatory mass or metastasis from the prostate was made. The mass failed to respond to a two-week course of ciprofloxacin. Bilateral orchidectomies were performed to remove the left epididymal mass and achieve an alternate form of hormonal control.

Histology revealed a poorly differentiated metastatic prostatic adenocarcinoma (PSA positive) to the left epididymis. There was no evidence of a primary testicular or epididymal tumor. The right testis and epididymis were normal. A bone scan confirmed bony metastasis.

## DISCUSSION

Epididymal metastasis from prostatic carcinoma is uncommon. It was first reported by Humphry [1] in 1944. When Wiebe et al [2] reported a second case in 1993 he found only 14 previous cases in the literature.

Most cases have been incidental findings following bilateral orchiectomies for metastatic prostatic carcinoma. Occasionally it may present as a solitary metastasis [3]. In this case it mimicked an inflammatory epididymal mass on account of the associated tenderness.

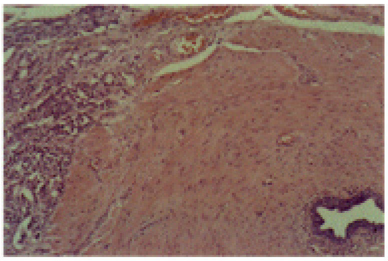

**FIGURE 1**. Prostatic adenocarcinoma infiltrating the efferent ductules of the testis/epididymal lobules. x200 Hematoxylin & Eosin stain.

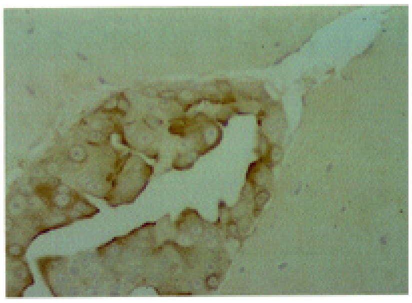

**FIGURE 2**. The tumor is positive with antibodies to Prostatic Specific Antigen (PSA). x400 Immunoperoxidase stain.

## CONCLUSION

Although uncommon, metastasis should be considered in the differential diagnosis of epididymal masses in patients with known prostate cancer.
